# Commercial Egg Replacers in Pound Cake Systems: A Comprehensive Analysis of Market Trends and Application

**DOI:** 10.3390/foods13020292

**Published:** 2024-01-17

**Authors:** Juliane Halm, Aylin W. Sahin, Laura Nyhan, Emanuele Zannini, Elke K. Arendt

**Affiliations:** 1 School of Food and Nutritional Sciences, University College Cork, T12 K8AF Cork, Ireland; juliane.halm@umail.ucc.ie (J.H.); aylin.sahin@ucc.ie (A.W.S.); lnyhan@ucc.ie (L.N.); e.zannini@ucc.ie (E.Z.); 2 Dipartimento di Biologia Ambientale, Sapienza Università di Roma, 00185 Rome, Italy; 3 APC Microbiome Ireland, University College Cork, T12 YT20 Cork, Ireland

**Keywords:** egg replacement, market research, plant-based cakes

## Abstract

Replacing eggs without influencing pound cakes’ texture, appearance, and taste is challenging. Ovalbumin, the major protein in egg white, contributes to the structures of cakes by providing SH Groups that form a firm gel during baking. However, there is a shift in the consumers’ behaviour regarding health, well-being, animal welfare standards, and environmental concerns. To meet upcoming trends and consumer needs, 102 egg replacement products were launched globally to the best of the authors’ knowledge, with 20 of them advertised as suitable for baking applications. Ten locally available commercial egg replacers with a range of protein contents were chosen and applied in a pound cake model system to evaluate their functionality by evaluating cake and cake batter quality. Three different categories of egg replacements were chosen: replacers containing no protein (R1–R3), a low amount of protein (1–10 g/100 g; R4–R5), and a high amount of protein (>10 g/100 g; R6–R10). Those were compared to three control cakes containing powdered whole egg, fresh egg, and liquid whole egg. All the analysed egg replacers significantly differed from the control cakes, including low-protein egg replacement R4. Despite R4 achieving the highest specific volume (1.63 ± 0.07 mL/g) and comparable texture values, none of the examined egg replacers compared favourably with the egg control cakes regarding appearance, physical and textural properties, and nutritional value.

## 1. Introduction

The demand for eggs and products derived from eggs in Europe is expected to grow by 20% until 2030 [1]. However, since the last decade, consumers’ behaviour has changed regarding health, well-being, and environmental concerns [2]. Several issues are directly related to the conventional production of eggs. For instance, there has been a higher awareness of animal welfare standards since banning cage systems in 2012 [3,4]. Another point is correlated with eggs being produced from animals, hence the occurrence of food safety scandals such as the Fipronil scandal in the European Union in 2017 [5] or the salmonella outbreak [6] in the United States in 2015. Also, high fluctuations in the prices of eggs due to outbreaks of avian influenza, causing bird losses, pose a problem for the industry [7]. Furthermore, environmental issues are posed by the egg industry, being responsible for about 10% of total livestock emissions [8]. As a result, there is a growing demand for egg alternatives originating from plants that replicate similar functionalities [9]. The global market for egg alternatives accounted for USD 1.5 billion in 2021, with an expected annual growth rate of 8.3% through 2031 [10]. From 2016 to 2021, 102 egg replacement products were launched globally [2]. There are various applications for egg alternatives, including mayonnaise, biscuits and cookies, noodles and pasta, products to simulate egg dishes (e.g., scrambled eggs), chocolates, and cakes [2]. 

To simulate eggs’ physicochemical, techno-functional and sensory attributes, it is essential to understand their complex composition and structure [11]. Eggs are large single cells that contain all important materials to contribute to the initial development of the embryo. Therefore, an egg is enriched with the necessary proteins, lipids, carbohydrates, vitamins, and minerals [11,12]. In summary, a whole hen’s egg contains 75% water, 12% protein, 12% lipids, and smaller amounts of carbohydrates, vitamins, and minerals [13]. Pound cake is a complex food system containing mainly eggs in addition to wheat flour, sugar, margarine, or butter, added in equal portions [14,15]. Broken down, the cake batter is divided into a continuous aqueous phase with dissolved sugars and proteins, hydrated flour particles, and margarine or butter emulsified in the liquid phase. The incorporation of air creates foam, consisting of air bubbles and small water cells, which are stabilised by the fat phase of the system [16]. Forming a stable protein network contributes to the required properties of pound cake. Egg protein is mainly responsible for the end product’s characteristics, such as microstructure, appearance, texture, and sensory attributes [17]. However, the batter preparation does not contribute to the protein network formation significantly. One reason is the presence of fat and the high sugar contents that restrict the gluten network development during mixing [18]. In contrast to the batter preparation, multiple covalent and SS bonds are formed during the baking process. Therefore, it is hypothesised that different proteins from the various ingredients in cake (flour and egg) interact with each other during baking, whereas ovalbumin is the most crucial factor regarding the development of SS and covalent bonds [14,19]. Furthermore, SS bonds are formed with protein from egg yolk and wheat flour through SH–SS exchange reactions between 78 and 84 °C [20]. Following higher temperatures (between 84 and 90 °C), the denaturation of ovalbumin allows the incorporation of other proteins from wheat flour, such as α- and γ-gliadin [21]. In addition, the LDL fraction of the egg yolk plasma is a strong emulsifier. The fraction contains proteins and phospholipids. These amphiphilic molecules contribute to surface activity [12]. Adding eggs to pound cake is vital in forming a stable protein network between all available protein sources, guaranteeing adequate cake quality.

Multiple studies focused on the replacement of eggs in bakery products. The egg alternatives, based on soy, wheat, whey, or hydrocolloids, had an inferior effect on the products and were not comparable to the egg-containing control cakes [22,23]. Furthermore, the performance of soy milk as a potential egg replacer was evaluated [24]. The addition of the emulsifier distilled glycerol monostearate led to promising results whereas adding lecithin resulted in a dark-coloured cake with a firm texture and low volume and sensory scores [25,26]. Other studies evaluated the influence of emulsifiers on whey protein as a possible egg replacement. Both could determine a positive effect of the added emulsifiers, although the final products did not match the quality of the control cakes [27,28]. Overall positive effects could be achieved by using lentil protein as a replacer in muffins and angel cake, excluding the hardness and chewiness parameter, which increased over storage [29]. Agrahar-Murugkar et al. (2016) could identify banana as an excellent egg replacer whereas adding chia seeds was less accepted by consumers [30]. However, chia seeds increase the protein, fat, and mineral content [31]. Aslan and Ertas (2020) added chickpea aquafaba in different concentrations [32]. The maximum addition of 50% aquafaba to the cakes had no effect on the cake quality.

The objective of this study was to evaluate a selection of egg replacers currently on the market in a pound cake system. Egg replacers were chosen based on their local availability while also selecting products that ranged in protein content in order to obtain samples representative of the egg replacer market. A variety of quality criteria were applied to determine their suitability in comparison to control cakes. It is hypothesised that replacing eggs with commercial egg replacers impacts the quality attributes of the baked good in comparison to the egg-based controls.

## 2. Materials and Methods

### 2.1. Market Analysis

A web search was performed to obtain an overview of the commercially available egg replacer suitable for baking. This search was focused on plant-based egg alternatives except for one whey-based egg replacer. The nutritional composition, protein sources, carbohydrate sources, usage of additives, claims on the product, country of origin, and prices of the different egg replacements were evaluated (performed in June 2023).

### 2.2. Ingredients

The pound cake was produced using biscuit flour (Odlums Group, Dublin, Ireland), sucrose (Siucra, Dublin, Ireland), salt (Glacia, UK), vegetable fat spread (Upfield Holdings B.V., Amsterdam, The Netherlands), whole egg powder (C1) (Igreca, Seiches sur le Loir, France), filtered tap water, and baking powder (Dr. Oetker, Bielefeld, Germany). During the trials, the whole egg powder was equally replaced with fresh eggs (C3) (Clóna, Clonakilty, Ireland) and pasteurised liquid whole eggs (C2) (O’Egg Free Range Eggs, Kells, Ireland) as additional controls. Additional controls were added to compare the functionality of commercial egg replacers to that of different egg products used by industry and regular consumers.

### 2.3. Egg Replacers

Ten egg replacers representing different compositions, which were specially advertised for bakery applications, were chosen for analysis. The commercial names and producers are not revealed here, and egg replacers were consecutively numbered. The ingredients of the commercially available egg replacers tested are listed in Table 1 according to the suppliers; the nutritional composition is given in Table 2. The information was taken from the labels of the commercial egg replacers. 

#### 2.3.1. Appearance of Ingredients

The appearance of the ingredients was investigated by taking pictures (Apple iPhone 11, Apple Inc., Cupertino, CA, USA). 

#### 2.3.2. Morphology of Ingredients

The ultrastructure of the cake crumb was investigated using scanning electron microscopy (SEM) to evaluate the structures of the added egg powder and egg replacement ingredients. Therefore, the egg powder and replacements were freeze-dried (SP Scientific, Warminster, PA, USA) and immobilised on an aluminium stub by using double-sided carbon tape. Afterwards, sputter-coating technique using palladium–gold (5 nm coat thickness) was applied. Samples were observed in a field emission scanning electron microscope with a working distance of 20 mm. SEM Control User Interface software, Version 5.21 (JEOL Technics Ltd., Tokyo, Japan), was used for taking images at an accelerating voltage of 5 kV.

### 2.4. Cake Batter Preparation

The recipes of the different cakes are illustrated in Table 3. Firstly, for the control cake (C1), water was added to the whole egg powder and the mixture was premixed using a Kenwood Chef (Kenwood, Havant, UK) equipped with a whisk mixing attachment (30 s, speed 6). Accordingly, the control cakes C2 and C3 were prepared by premixing the fresh eggs (C2) and pasteurised liquid eggs (C3). The egg replacer pound cakes were prepared following the supplier’s instructions, adding the same premixing step used for the control cakes. Following, sucrose and salt were added to the egg mix or the prepared egg replacers. The combined ingredients were mixed using a Kenwood Chef (Kenwood, Havant, UK) equipped with a whisk mixing attachment (60 s, speed 4.5). After one minute, the speed was raised (60 s, speed 5). Next, biscuit flour and baking powder were added. Finally, the melted shortening was slowly incorporated into the mixture during the second mixing process (60 s, speed 4; 60 s, speed 3).

#### 2.4.1. Batter Changes during Baking: Micro-Baking

Changes in visco-elastic properties of the cake batter during heating were determined using the micro-baking test reported by Schirmer et al. (2012) using a Rheometer Physica MCR 301 (Anton Parr GmbH, Ostfildern, Germany) equipped with a plate–plate (probe diameter: 50 mm) measurement system [33]. The complex modulus (G*) and the temperature (T_i_) at the curve’s inflexion point were determined.

#### 2.4.2. Cake Batter Viscosity

The viscosity properties of the cake batter were studied using a Rheometer Physica MCR 301 (Anton Parr GmbH, Ostfildern, Germany). The cake batter was transferred into the concentric cylinder (CC) measurement system and filled to the level mark, and a measurement probe with a diameter of 26.7 mm was used. The apparent viscosity at a shear rate of 10 s^−1^ and a temperature of 20 °C was determined.

### 2.5. Cake Preparation

250 g of the cake batter was transferred into a baking pan (height: 58.14 mm; top length: 149.62 mm; bottom length: 138.46 mm; top width: 85.16 mm, bottom wideness: 75.22 mm). The batters were baked in a deck oven (MIWE condo, Arnstein, Germany) preheated to 180 °C for 36 min. After baking, the cakes were cooled at room temperature for two hours for further analysis. 

#### 2.5.1. Bake Loss

The bake loss (%) during baking was determined through weight difference before and after baking. 

#### 2.5.2. Specific Volume

The specific volume was determined using a VolScan Profiler (Stable Micro Systems, Godalming, UK).

#### 2.5.3. Crumb Texture 

The crumb texture of the cakes was determined by using texture profile analysis (TPA) with the TA-XT2i texture analyser (Stable Micro Systems, Godalming, UK), attached with a flat-ended cylindrical aluminium probe (diameter: 25 mm) and a TA-90 platform. Cakes were cut into 25 mm thick slices, and end slices were not considered for analysis, resulting in four slices per cake. A two-compression test with a pre-test speed of 1.0 mm/s, a test speed of 5.0 mm/s, a post-test speed of 10 mm/s, and a travel distance of 10 mm was applied. Hardness (peak force during the first compression cycle), springiness (distance from the start to the maximum of the second peak), cohesiveness (ratio of the positive force area during the second compression to that during the first compression), chewiness (negative force area for the first bite), resilience (ratio of the energy of both peaks), and adhesiveness (peak area force of the negative peak) were evaluated [34]. 

#### 2.5.4. Crumb Structure

Cakes were cut into 25 mm thick slices, and end slices were not considered for analysis, resulting in four slices per cake. The C-Cell imaging system (Calibre Control International Ltd., Warrington, UK) was used for crumb structure analysis, including the number of cells, the number of holes, the cell elongation, and the cell diameter. 

#### 2.5.5. Crust and Crumb Colour

The colour of the crust and crumb was measured using a Colorimeter CR-400 (Konica Minolta, Osaka, Japan). The CIE L* a* b* colour system was applied, and the colour difference compared to the control cake C1 (whole egg powder) was calculated using the Scofield equation. The crust colour of each cake (one replicate) was measured by taking ten measurements at ten different areas of the top of the cake. The crumb colour of a cake was determined through five measurements per cake slice for four slices per cake in summary.

$$∆E=\sqrt{{\left(∆L\right)}^{2}+{\left(∆a\right)}^{2}+{\left(∆b\right)}^{2}}$$

#### 2.5.6. Water Activity

The water activity was measured using the Aqua Lab water activity meter (AQUA LAB GmbH & Co.KG, Höhr-Grenzhausen, Germany). A part of the cake crumb was placed in an Aqua Lab measurement cup, the cup was closed by a lid, and the sample was equilibrated for five minutes before starting the measurement. 

### 2.6. Statistical Analysis

All analyses were performed in triplicates. The cakes were prepared in duplicate on three different days. A variance analysis (one-way ANOVA, *p* ≤ 0.05, Tukey test) was performed using OriginPro 2023 (version: 10.0.0.154). Additionally, a principal component analysis (PCA) was performed in order to investigate the influence of the type of egg replacement and the cake properties determined. 

## 3. Results

### 3.1. Market Analysis

**Figure 1:**
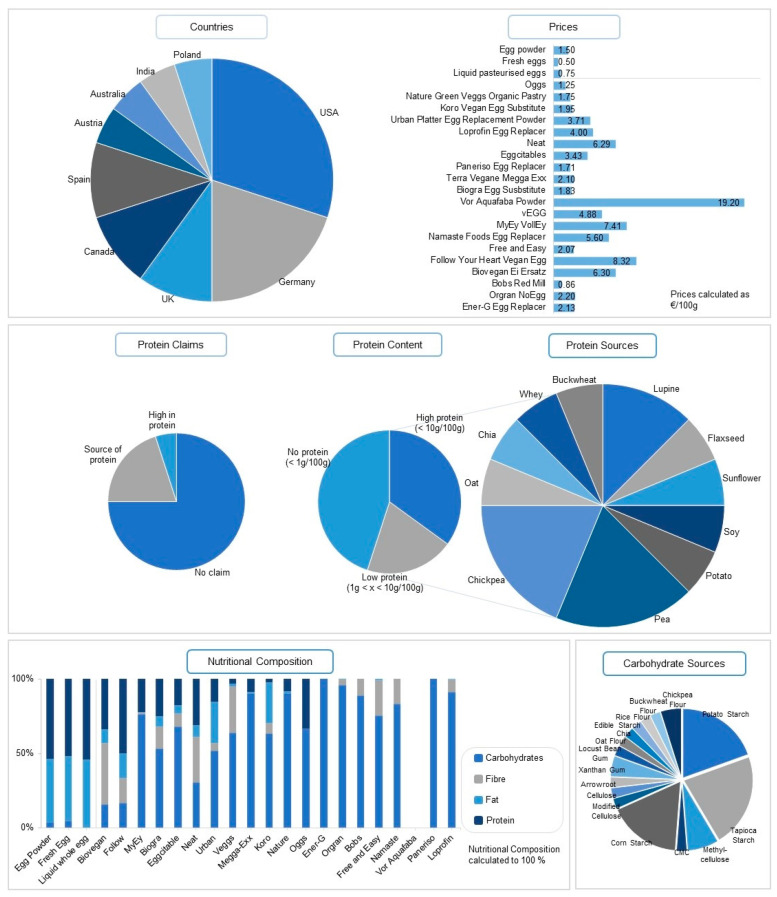
Overview of commercially available plant-based egg replacements (last updated 10 May 2023), including one whey-based egg alternative.

This market analysis focused on egg replacers specifically suitable and marketed for the preparation of cakes. Figure 1 summarises the general trends of the accessible egg alternatives. Currently, 20 products related to this purpose are available on the global market for consumers (sourced data update: June 2023). Most of the egg alternatives are sold as powders whereas only one is marketed in a liquid form. This correlates with market analysis from 2019, where the powder segment was the most dominant sector, accounting for USD 815.4 million [35]. More than fifty percent of the egg replacers suitable for baking are produced in North America (US, Canada) and Germany. Therefore, North America’s general market for egg alternatives is the most dominant, estimated at 47.8% globally. After the USA, Germany produces a high amount of vegan egg replacements. To summarise the main market trends, the claims of the different products were evaluated. The majority of egg alternatives claim to be “vegan” (75.0%), “gluten-free” (55.0%), and “non-GMO” (45.0%). Claims such as “soy-free” (25.0%), “no cholesterol” (25.0%), and “kosher” (20.0%) are also frequently used by manufacturers. 

Most egg replacers contain any protein (45.0%) or very low protein amounts (20.0%) and are nutritionally not comparable to commercial egg powder. Egg products contain mostly proteins and fat while vegan egg substitutes provide a nutritional profile rich in carbohydrates and fibre. This is also reflected in the information provided by the manufacturers. The most abundant protein sources for the egg replacers are pea (25.0%), chickpea (25.0%) and lupine (16.7%), with all of them being categorised as pulses. The nutritional composition of most of the egg replacers on the market comprises a mixture of starches and hydrocolloids, neglecting the addition of any protein. Tapioca (45.0%), potato (40.0%), and corn starch (35.0%) are the most abundant carbohydrate sources used in vegan egg replacers. Hydrocolloids that enhance plant-based proteins’ natural foaming, thickening, and emulsifying properties are added in various formulations. The hydrocolloids methylcellulose, xanthan gum, carboxymethyl cellulose, cellulose, modified cellulose, and locust bean gum are applied in the egg replacers present in this market analysis. 

### 3.2. Cake Batter Properties

#### 3.2.1. Structure Changes during Heating

The transition temperature T_i_ of the cake batter from the liquid state to the solid state was determined by using the inflection point of G*, also considered as correlating to the stiffness of the cake batter. The results are illustrated in Table 4. The T_i_ of the whole dry egg (C1) cake batter was 82.47 ± 0.39 °C, showing no significant differences from the fresh egg cake batter (C2) (82.18 ± 0.83 °C) or the batters containing R10 (83.97 ± 0.97 °C), R8 (82.87 ± 0.39 °C), and egg replacer R1 (81.12 ± 0.71 °C). The lowest temperature at the inflection point (80.67 ± 0.63 °C) was achieved by C3 (pasteurised liquid egg), which was not comparable to the remaining analysed cake batters. However, a significantly higher T_i_ was obtained by incorporating R7 (86.72 ± 0.27 °C), R4 (86.50 ± 0.49 °C), and R6 (86.30 ± 0.89 °C) into the cake batter.

#### 3.2.2. Cake Batter Viscosity

The apparent viscosity of the cake batters is displayed in Table 4. The cake batter with dry whole egg (C1) resulted in an average apparent viscosity of 18.08 ± 1.01 Pa∙s, while fresh egg (C2) and liquid pasteurised egg (C3) showed significantly lower values, 10.40 ± 1.38 Pa∙s and 10.71 ± 0.66 Pa∙s, respectively. The only egg replacer achieving a comparable result to C1 was R8 (18.09 ± 1.21 Pa∙s). The remaining egg replacers were significantly different from C1. Incorporating R1 (13.92 ± 1.85 Pa∙s), R2 (11.87 ± 1.14 Pa∙s), R5 (11.33 ± 1.12 Pa∙s), and R9 (12.43 ± 0.84 Pa∙s) into the recipe led to an apparent viscosity comparable to C2 and C3 batter samples. Adding R4 to the batter mixture resulted in the most viscous cake batter (83.34 ± 11.79 Pa∙s).

### 3.3. Egg Replacer Properties

#### 3.3.1. Appearance of Ingredients

**Figure 2:**
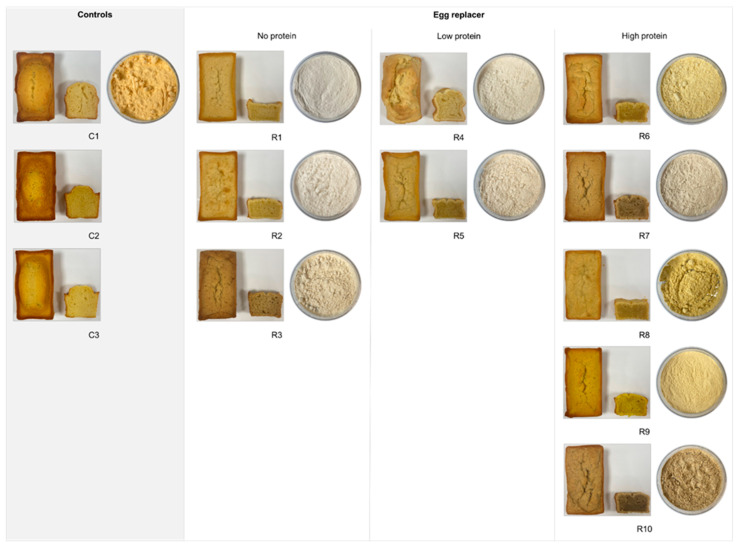
Appearance of the pound cakes and the egg replacer ingredients. C1 represents whole egg powder, C2 fresh eggs, and C3 liquid pasteurised eggs. R1–R10 illustrate the other egg replacers used in the pound cake recipe.

The appearance of each egg replacer ingredient is shown in Figure 2. The colours of the ingredients vary from white (R1, R2, R4), beige (R3, R5, R7), and yellow (R6, R8, R9) to brown (R10). The textures of the ingredients are powdered and clumpy, except for the smooth texture of R1 and R9. 

#### 3.3.2. Morphology of Ingredients

**Figure 3:**
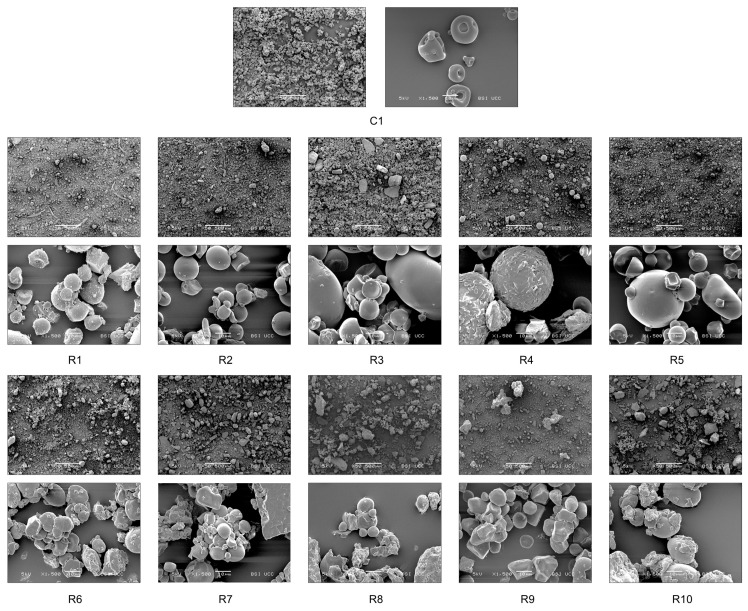
SEM micrographs of applied ingredients and egg replacers, captured using a scanning electron microscope (SEM) with magnifications of ×50 and ×1500. C1 represents whole egg powder, C2 fresh eggs, and C3 liquid pasteurised eggs. R1–R10 illustrate the other egg replacers used in the pound cake recipe.

Scanning electron micrographs (Figure 3) illustrate the differences in ingredient morphology with magnifications of ×50 and ×1500. Compared to the dry whole egg, most egg replacers are composed of intact starch molecules/granules. Soft-rounded globular protein structures are visible in the egg powder R5, containing pea protein; R8, containing sweet lupine protein; and R9, containing both lupine and pea protein. Pulses like sweet lupine and pea contain globular proteins like albumin. The shape of those proteins resembles densely packed spheres as visible in Figure 3. The shape occurs due to intermolecular forces such as hydrophobic effects, hydrogen bonding, electrostatic forces, van der Waals forces, and disulfide bonds [36]. The egg replacer R4 displays a different morphology. Large micelles with a rough surface are distributed throughout the product. However, egg alternatives R6, R7, R8, and R10 are characterised by sharp-edged structures compared to the evenly dispersed starch-based replacers. Additionally, distinct chunky particles are visible in the micrographs of R7 and R3.

### 3.4. Cake Properties

#### 3.4.1. Bake Loss 

Bake loss determines the loss of moisture and volatile components during baking. The results are demonstrated in Table 5. The bake loss of the whole dry egg control cake (C1) was 9.39 ± 0.61%. Comparable results were observed in cakes with C2 (9.38 ± 0.41%) and egg replacers R2 (10.02 ± 0.33%), R6 (9.79 ± 0.09%), and R10 (8.78 ± 0.27%). The highest bake loss was obtained by incorporating egg replacer R3 (11.68 ± 0.41%). Egg replacement R4 achieved the lowest bake loss with 6.93 ± 0.41%. Applying pasteurised liquid egg (C3) to the pound cake recipe resulted in a value not comparable to the other control cakes (10.79 ± 0.31%). However, the egg replacers R1 (11.26 ± 0.49%), R2 (10.02 ± 0.33%), R5 (10.88 ± 0.19%), R7 (10.59 ± 0.23%), R8 (10.21 ± 0.68%), and R9 (11.16 ± 0.27%) result in comparable results to C3.

#### 3.4.2. Specific Volume

**Figure 4:**
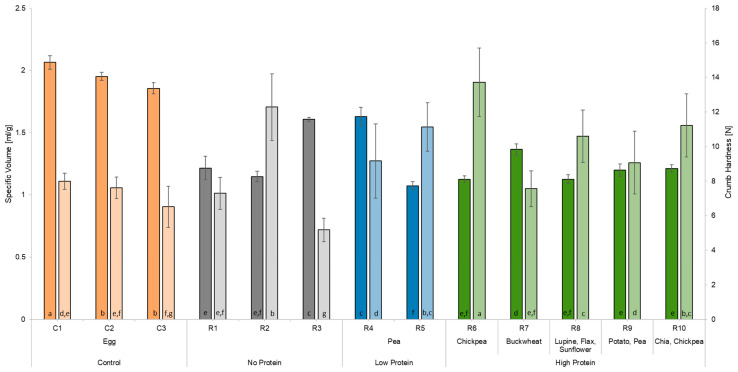
Specific volume and crumb hardness of the different cakes produced. C1 represents whole egg powder, C2 fresh eggs, and C3 liquid pasteurised eggs. R1–R10 illustrate the other egg replacers used in the pound cake recipe. Values are given as the average ± standard deviations. No significant difference occurred between values in the same column with the same lowercase letter (*p* < 0.05).

Figure 4 displays the values for the specific volume of the different cakes. The control cake C1 showed the highest specific volume with 2.07 ± 0.05 mL/g, followed by the other control cakes C2 (1.95 ± 0.03 mL/g) and C3 (1.86 ± 0.04 mL/g). The egg replacers reaching specific volumes closest to the control cakes were R4 (1.63 ± 0.07 mL/g) and R3 (1.61 ± 0.02 mL/g). The lowest specific volume was obtained by using R5 (1.07 ± 0.03 mL/g), a starch-based egg replacer with the addition of pea protein, followed by the two egg replacements R8 (1.13 ± 0.04 mL/g), a sweet lupine flour-based replacement, and R6 (1.13 ± 0.03 mL/g), based on corn starch with the addition of chickpea flour. As Figure 4 shows, no significant differences appear between the remaining egg replacers.

#### 3.4.3. Textural Analysis

In relation to the specific volume, the crumb hardness is displayed in Figure 4. The control cake C1 is characterised by a soft crumb texture (7.99 ± 0.47 N), and a similar softness was determined in cake crumbs containing control C2 (7.61 ± 0.62 N) and egg replacers R1 (7.29 ± 0.93 N), R4 (9.16 ± 2.16 N), R7 (7.56 ± 1.04 N), and R9 (9.06 ± 1.82 N). C3, as a control, showed a significantly lower crumb hardness (6.51 ± 1.19 N) compared to C1. The egg replacers containing chickpeas (R6: 13.72 ± 1.99 N; R10: 11.22 ± 1.83 N) had a denser crumb texture, significantly different to that of the control cake C1. In addition to the hardness, the parameters of springiness, cohesiveness, chewiness, resilience, and adhesiveness of the cake crumb were determined (Table 5). The characteristics of the cake crumb texture including those of C1 were high springiness (0.91 ± 0.01), high cohesiveness (0.62 ± 0.01), moderate chewiness (4.55 ± 0.30 J), high resilience (0.24 ± 0.01), and low adhesiveness (−0.01 ± 0.00 J). The egg replacer most comparable to C1 was R10, with a springiness of 0.88 ± 0.02, a chewiness of 4.21 ± 0.82 J, and a gumminess of 4.80 ± 0.86. However, R10 had a significantly harder crumb. R6 also showed high similarities to C1 regarding chewiness (4.29 ± 0.89 J) and resilience (0.20 ± 0.17). The cohesiveness was the only attribute not matched by any commercial egg replacers. 

#### 3.4.4. Crumb Structure

The crumb structure analysis is presented in Table 6. The control cake C1 showed the highest number of cells (3099 ± 259). A similar result was achieved by replacing the whole egg powder with replacer R4 (3064 ± 238). Using the other egg replacers resulted in a deficient number of cells, with R8 (1403 ± 159) and R5 (1455 ± 131) having the lowest values. The other control cakes differed from C1 and were not comparable, with C2 (2798 ± 263) displaying a higher number of cells than C3 (2520 ± 190). None of the egg replacers were similar to the controls. The highest number of holes were detected in C1 (2.72 ± 1.35) and R4 (5.23 ± 2.03) whereas the lowest numbers of holes were found in the egg replacement cakes R5 (0.26 ± 0.51) and R3 (0.35 ± 0.41). The results for the cell diameter showed no significant differences from the control cakes C1 (1.91 ± 0.20), C2 (1.89 ± 0.14), and C3 (2.12 ± 0.16). The average cell elongation represents another parameter important for characterising the crumb structure. It is defined as the ratio of the diameter in horizontal (x) and vertical (y) directions. In summary, an ideal round-shaped cell would be represented by a value of 1.00. The control cake C1 with an average cell elongation of 1.48 ± 0.01 did not differ significantly from the cakes including the egg replacements. The only cake significantly different in the cell elongation parameter was C3 (1.65 ± 0.28), displaying the highest value. The cakes containing R1 (1.44 ± 0.02) and R7 (1.44 ± 0.02) showed the closest value to ideal round-shaped cells. In contrast, the cake produced from egg replacement R4 (1.58 ± 0.03) resulted in an increase in elongation. 

#### 3.4.5. Crust and Crumb Colour

For comparing the crust and crumb colour of the different cakes, the ΔE values were calculated, considering the L*, a*, and b* values. The calculated values are displayed in Table 6. The higher the ΔE value was, the more significant the difference from the whole dry egg control pound cake (C1) recipe was. The incorporation of R3 resulted in the most important difference in the crust colour (ΔE 10.85 ± 1.41). The lowest difference was achieved by adding R6 (ΔE 1.57 ± 1.95), R7 (ΔE 2.26 ± 1.63), R1 (ΔE 2.62 ± 2.72), R2 (ΔE 2.70 ± 1.53), and R4 (ΔE 2.85 ± 0.99). Compared to the remaining control cakes C2 (ΔE 4.10 ± 0.96) and C3 (ΔE 2.14 ± 2.40), no significant differences appeared in the crust colour of the egg replacement cakes. Regarding the crumb colour, the cake containing R9 showed a minor difference (ΔE 3.09 ± 2.98). No significant differences to the crumb colour of C1, appeared in the control cakes C2 (ΔE 3.04 ± 0.61) and C3 (ΔE 2.28 ± 1.81) and, additionally, the egg replacer cakes R4 (ΔE 4.57 ± 1.33) and R9. The highest differences in colour were shown by the application of R7 (ΔE 18.01 ± 0.20), R5 (ΔE 18.36 ± 0.87), and R10 (ΔE 29.10 ± 2.13). 

#### 3.4.6. Water Activity

Most of the egg replacers’ water activity (a_w_) differs significantly from that of the powdered whole egg (C1) control cake. The only egg replacer not significantly different from C1 (0.765 ± 0.064) was R2 (0.809 ± 0.040). The a_w_-values of the other cakes (listed in Table 5) showed no significant differences and were not comparable to the control cake C1, but were comparable to control cakes C2 and C3.

## 4. Discussion

This study revealed the decisive function of eggs in a pound cake system and the effects of commercially available egg replacers on batter and cake quality, as well as on nutritional value.

A recent survey on the current market situation stated that manufacturers face challenges developing plant-based eggs, making most of the available products purely starch-based. In conclusion, most of the problems lie within replicating the functionalities and taste of conventional eggs, the high ingredients costs, and the limited capacity of start-ups to scale the product [37]. Starch-based egg replacers fail to replicate the high nutritional value of eggs. In contrast, applying plant-protein-based substitutes (soy, whey, lentil) results in a low consumer acceptance, considering the low volume of the cakes, inappropriate texture, and sensory scores [22,23,24,25,26]. Promising results were found upon applying lentil protein as an egg replacement in muffins and angel cake, with minor concerns about the texture of those bakery products [29]. Other studies claimed chickpea aquafabe as a fitting egg replacement. Treatment with citric acid and table salt resulted in a remarkable similarity to baked products containing egg whites [38]. Another study added lentil protein to a mixture of aquafabe and citric acid and achieved acceptable cakes [39]. A potential approach for the substitution of eggs in baked goods involves the usage of plant milk. In a recent study, 50% of eggs were successfully replaced with soy or lupin milk [40]. Europe’s trending vegan food market [2] contributes to multiple European countries selling and developing vegan egg replacements such as the UK, Spain, Austria, and Poland. Product claims on egg replacer packaging serve as advertisements for the advantages of using plant-based egg alternatives such as their lower environmental impact, considering that many consumers still perceive conventional eggs as a good option [37]. Additionally, egg replacers are considered healthy by recent surveys, providing essential minerals, vitamins, and a high amount of fibre [41]. However, most of the analysed egg replacers display a poor nutritional value not comparable to eggs, with only 10% of the available products reaching a high in protein claim (according to EU, Regulation (EC) No 1924/2006; and Canadian regulations, B.01.513 Food and Drug Regulations (FDR)). Moreover, the protein quality of egg replacers is often inferior to that of eggs as these replacers contain pulses that contain low amounts of sulphur amino acids. To overcome this problems, adding cereal proteins containing natural sources of sulphur amino acids and hydrocolloids is suggested [12]. 

**Figure 5:**
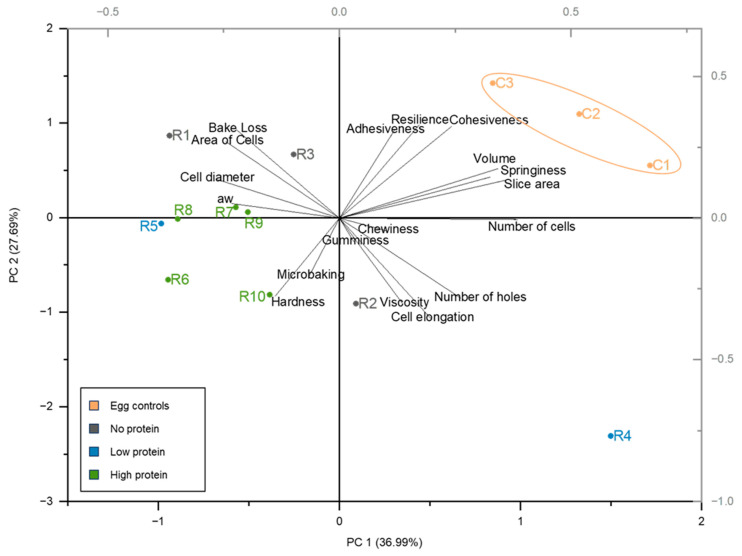
Principal component analysis (PCA) plot of the determined cake attributes (except crust and crumb colour). C1 represents whole egg powder, C2 fresh eggs, and C3 liquid pasteurised eggs. R1–R10 illustrate the other egg replacers used in the pound cake recipe.

The quality of a cake is directly related to the properties of the cake batter. In Figure 5, the different attributes of the cake samples are plotted in a principal component analysis (PCA). In the PCA, a direct correlation occurs between the analysed parameters of cell elongation and the number of holes. Correlation analysis reveals a direct correlation between the viscosity of the cake batter and the number of holes (*p*-value: 0.000843; r-value: 0.80). Hence, the batter viscosity influenced the expansion and stability of air bubbles in the batter throughout the baking process [28]. In less viscous cake batter, the carbon dioxide can evolve, releasing the water vapour from the system. Comparatively, highly viscous cake batters retain more air bubbles, leading to more holes [28]. On the other hand, the PCA displays no positive or negative correlation between the viscosity and the texture properties, resulting in a limited influence of the cake batter’s viscosity on the overall textural quality of the product. It has been reported that hydrocolloids prevent structural collapse by increasing the viscosity of the batter, particularly xanthan gum, a compound whose high molecular weight correlates with the radius of gyration and water hydration capacity, leading to a high batter viscosity [28,42,43,44]. A clear trend involving the usage of hydrocolloids and the occurrence of higher viscosity is not visible in the results. However, it is possible that the application of R4 resulted in the most viscous batter due to the included xanthan gum. Contradictory to this, the other egg replacers containing xanthan gum (R2, R9) are characterised by a low viscous batter, even with the presence of salt-based ingredients (cream of tartar, rock salt, black salt) that are known to promote the association and gelation properties of xanthan gum [45]. A direct correlation was observed between the water content and the viscosity (*p*-value: 0.00809; r-value: 0.77), resulting in the assumption that the water level has a stronger influence on the viscosity than the included hydrocolloids. For better comparability and the estimation of the effects of the ingredients, the same amount of water should be added instead of following the supplier’s instructions. 

Additionally, the structural changes during the baking process of the cake batter were analysed. The lowest structure setting temperatures occurred in the control cakes, especially with fresh eggs (C3). This can be explained through the cake structure formation. During baking, the structure sets due to the gelatinisation of starches and egg protein coagulation [15]. Ovalbumin, the most abundant protein in egg white, completes the heat setting of the cake batter to a solid cake foam at 85 °C [15]. Comparable results for the egg replacers were only achieved by incorporating R1, R8, and R10, implying higher structure-setting temperatures for the remaining egg replacers due to the lack of egg proteins. This hypothesis is supported by the fact that plant proteins, such as pea protein isolate (88.9–94.5 °C), used in R4, R5, and R9, have a higher general denaturation temperature than egg proteins [46]. Hence, improving the quality of egg-replaced cakes could be achieved by lengthening the baking time or increasing the baking temperature. Another factor that influences the ability of structure setting during the heating process is the presence of hydrocolloids. The highest determined temperatures for the transition to a solid cake structure can be found in cakes including gelling agents like xanthan gum (R4, R6, R9). For the gelatinisation of starch, the water in the pound cake system is required, resulting in the swelling of starch granules [47]. The degree of gelatinisation is determined through the availability of water [14]. Since xanthan gum hydrates in cold solutions, water availability could be limited in egg replacers including this hydrocolloid [44]. In contrast, egg replacements, including the hydrocolloid methylcellulose (R1) and locust bean gum (R8), without additional xanthan gum, displayed lower structure-setting temperatures in this study. One explanation could be that locust bean gum requires heat to be soluble in water, and the gelation mechanism of methylcellulose is caused by the hydrophobic-induced association of methyl groups in methylcellulose at high temperatures. Therefore, they cannot absorb water during the mixing process, displaying no direct competition for the starch granules during cold temperatures, leaving enough available water for the baking process [48].

Nevertheless, the parameter specific volume displays the most noticeable difference when comparing the control pound cakes to the egg replacer cakes. The interaction of egg proteins and the gluten network form a strong thermal gel that strengthens the cake structure during baking [15,49,50]. Between the different control cakes C1, C2, and C3, significant differences in the specific volume are visible. This can be explained by the influence of the spray drying process used to manufacture whole egg powder (C1). High temperatures and pressure conditions during the drying process result in the denaturisation of the albumin fraction of the egg and changes in the protein conformation, positively affecting the emulsion stability and gel-forming capacity after heating, hence providing a higher specific volume in the cake [51]. The egg replacers achieving the highest specific volume are R4 and R3, containing no or minimal protein contents. The high specific volume of R4 is due to mono- and diglycerides, which delay starch gelatinisation and structure settings in cakes and allow for longer rising times [43]. Moreover, the formation of amylose–lipid complexes between certain emulsifiers and the amylose in wheat flour starch influences the starch gelatinisation during baking and results in a soft crumb, which correlates negatively with the specific volume (*p*-value: 0.0014; r-value: −0.68) [52]. R3 resulted in a high specific volume, most likely due to containing baking soda and psyllium husk fibre. Psyllium husk fibre is a highly branched acidic arabinoxylan with a high molecular weight (~1500 kDa), used as a stabiliser and gel former in bakery products [53]. However, arabinoxylan can beneficially influence the cake’s quality by increasing the batter’s viscosity, improving the water binding capacity and stabilising the expansion of gas cells during the baking process, resulting in a higher specific volume. Contradictory, it has been found that the increase in the batter viscosity above a critical level restricts the expansion of gas cells, directly relating to small volumes in cakes [54]. This could also relate to the collapsed structure that most of the egg replacement cakes display. Another explanation for the collapsed structure of the egg replacer cakes could be the restricted starch gelatinisation due to the competition for water by high-fibre ingredients, additional starches, and hydrocolloids [55]. The lowest specific volume was found in egg replacers, containing the hydrocolloids guar gum, locust bean gum, and xanthan gum (R2, R5, R8, R9), potentially due to the high molecular weights of the hydrocolloids creating overly strong interactions and competing with the present water in the system, thus preventing the formation of a stable cake network [44,56]. Horstmann et al. (2018) could determine similar results by applying those hydrocolloids in a bread system. It was hypothesised that the high molecular weights of the hydrocolloids create overly strong interactions, subsequently competing over the present water in the system that prevents the formation of a stable cake network.

Similar to other published studies, a negative correlation between specific volume and hardness of the cake was observed (*p*-value: 0.0014; r-value: −0.68). Cakes with a softer crumb structure mainly contained starch-based egg replacers, with some containing psyllium, relating to the previously stated theory that adding arabinoxylans improves cake quality. In contrast, no clear trend was discernible for the cakes with the hardest crumb structure (R6, R2, R5), which were equally and mainly starch-based. Hence, it can be assumed that the composition of the applied starch-based egg replacers provides optimal cake quality. The egg replacers displaying a comparable hardness value to that of C1 contain xanthan gum (R4, R9). This effect can be linked to the negatively charged surface of xanthan gum, creating repelling forces that restrict the starch granules from swelling further and retard the leaching of amylose, resulting in reduced retrograded amylose in the cake [44]. Accordingly, choosing the right hydrocolloid and protein source to simulate the textural properties of egg-based cakes is one of the general concerns for all upcoming egg replacer developments. Chewiness is a measure of the energy required for chewing semi-solid foods and can often be compared to the hardness parameter in egg-replaced cakes [25,30]. Comparable results to the egg powder cake (C1) were achieved by R5, R6, and R10. Similarities in the composition of the egg replacements lie within the addition of hydrocolloids in R5 and R6. On the other hand, R10 contains Chia as a natural hydrocolloid, and this enhances the cake’s structure by inhibiting the kinetics of amylopectin retrogradation and the mucilage, influencing the water holding capacity and the protein network formation [57]. Furthermore, Chia influences the texture attributes cohesiveness, springiness, and resilience, which codetermine the quality characteristics of pound cake. Those properties are indicators of the internal resistance of food to compression and the development of a three-dimensional protein network [27]. None of the egg replacer cakes correspond to the cohesiveness values of the control cakes, implying the possibility of product damage during the production process and the formation of an unstable network [22]. A high springiness value is desired for cakes and indicates a fresh, aerated, and elastic end product [58]. This attribute was achieved by R4 and R10, both containing legumes as protein sources. Legume proteins form gels through electrostatic interactions, hydrophobic interactions, and hydrogen bonds whereas disulfide bonds do not contribute to forming a gel-like structure [59]. As a result, the electrostatic interactions significantly positively influence texture properties like hardness and, notably, springiness [59].

Extremely high and shallow bake loss values are typical for exceptionally moist and dry cakes, respectively, while both extremes can have a significant impact on the quality of the product [22]. The egg replacer R3, having the highest bake loss, contains psyllium husk fibre. Geera et al. (2011) could determine a fibre- and gum-based egg replacer as relatively incapable of binding water during the heating process, compared with other egg replacers and 100% egg formulations [22]. For the egg replacement R4, it could be argued that the included starch and hydrocolloid (xanthan gum), combined with less water added to the recipe, resulted in the lowest bake loss due to the higher water holding capacity [60]. Another egg replacement displaying a significantly low bake loss is R10, containing chia. Chia gel was found to have a high water holding capacity compared to hydrocolloids like guar gum and other protein isolates (lupine, cowpea, winged bean) due to the high amount of fibre and protein within the ingredient [61].

The overall appearance of the final cake products plays a significant role in meeting the consumer’s needs and increasing product acceptance. Figure 2 shows a major difference between the control and egg replacement cakes. Additionally, differences in the colour of the egg replacer products are visible and calculated using the Scofield equation (Table 6). The crust colour is formed through non-enzymatic chemical reactions involving the Maillard reaction and caramelisation, reactions that are highly influenced by pH and water activity (a_w_ [15,49,50]. Regarding the a_w_ results, there are no significant differences between the egg replacers and the control cakes, except for R2 displaying a low a_w_. Those results were comparable with the crust colour of C1. It was also reported that a higher protein content promotes browning through the Maillard reaction [26]. However, this observation seems to be only partially true, as the high-protein egg replacers differ more from the control cake than the no- and low-protein egg replacers. The crust colour primarily depended on the colour of the products, with the only significant differences pronounced in R3 and R10 (Figure 2, Table 6). Both egg replacers contain high-fibre ingredients like psyllium husk and chia, with reports claiming that adding chia to pound cakes results in a darker product colour due to the darker colour of the ingredient [62]. This effect is particularly striking when looking at the results of the crumb colour analysis, with the most comparable replacer being R9. The added turmeric powder provides a yellow colour to the egg replacer powder, tinting the cake batter and the resulting product after baking. Accordingly, turmeric powder might be a suitable alternative to replicate the orange–yellowish colour of the egg yolk.

The cake crumb characterisation was accomplished through image analysis. Except for the slice area and the number of cells, which are directly related to the cake’s specific volume, no significant differences seem to appear. The more air is incorporated into the batter during mixing and held in the batter by functional ingredients, the higher is the number of final cells observed in the cell structure. During the baking process, water evaporates, and the vapour is entrapped in the already existing air cells. The more air cells are present in the batter, the higher the amount of water vapour being entrapped is, which increases the internal pressure, resulting in a leavening effect (supported by the reaction of baking powder). The cell diameter indicates, firstly, how resistant the cake batter is towards extension. A high resistance limits the expansion of the batter and hence causes a small diameter (R2, R4). Secondly, the cell diameter is influenced by the stretchability of the batter, which avoids the collapse of air cells leading to holes [63]. It is worth noting that egg replacer R4 is the most comparable to the control cakes without considering cell elongation. The parameter cell elongation is directly related to the viscosity of the cake batter (*p*-value:0.000472; r-value: 0.75). A cell elongation of 1 represents a circle structure while values higher or lower than 1 indicate an elongation of the cells. All cakes showed cells vertically elongated, a phenomenon caused by the internal pressure in the batter during leavening in the oven. An ideal round shape of the cells can only be achieved if the internal pressure is equally released in all dimensions. Due to the tin walls, all cakes rise vertically during baking, causing a cell elongation [63]. This explains the higher cell elongation in R4 and R10, where the high viscosity of the cake batter restricted the even expansion of the cake cells, resulting in elongated cells that were less comparable to the control cakes C1 and C2. 

## 5. Conclusions

Recent shifts in consumer behaviour are being caused by a growing concern for health, well-being, animal welfare standards, and the environment. As a response to these changing trends and consumer demands, manufacturers have brought 20 different egg replacers to the market, suitable for baking applications. Different approaches regarding the compositions of the egg replacers were used to face the challenges that arise with replacing eggs in a pound cake system. The ten analysed egg replacements were sorted into three different categories, displaying egg replacements with no protein, a low amount of protein (1–10 g/100 g), and a high amount of protein (>10 g/100 g) and were compared to three different control cakes including powdered whole egg, fresh egg, and liquid whole egg. Applying a low-protein egg replacer, consisting of a corn-starch-based alternative, with the addition of mono- and diglycerides of fatty acids as emulsifiers, xanthan gum as a hydrocolloid, and pea as the protein source led to the most comparable results, especially regarding specific volume, textural properties, and colour. The added emulsifier was suspected to positively influence the results due to the replication of the emulsifying characteristics of the egg yolk. Psyllium husk fibre was another ingredient that positively affected textural properties, with its incorporation resulting in a softer crumb texture in one of the no-protein egg alternatives and one of the high-protein egg alternatives. Hydrocolloids, like xanthan gum, positively influenced the cake batter’s viscosity and texture properties, especially hardness. By contrast, they restricted the structure setting of the cake and inhibited the formation of a high specific volume as a direct competitor for the water in the system. Hot-swelling hydrocolloids (methylcellulose, guar gum, locust bean gum) performed better regarding the structure setting properties of the cakes. However, the nutritional composition of the analysed egg replacers could not match the positive attributes of eggs, particularly the high protein content and the even distribution of essential amino acids throughout the egg protein. According to the performed market research on the available egg replacers for baking purposes, this is a recurring issue. Additionally, market research has shown that egg replacers cannot match the functionality of conventional eggs in terms of volume, taste, and texture, which reflects the findings of the current study. On the other hand, egg alternatives containing higher amounts of plant-based proteins performed poorly in simulating the egg proteins’ characteristics during the baking process. Therefore, a new approach for developing egg replacements must be taken into consideration, using ingredients that provide the nutritional and techno-functional properties of eggs in a pound cake system. Limitations that need to be considered for the results of this research are the small sample size and the application in a controlled baking environment. Future research should focus on using more samples under different baking conditions, replicating the cake production in the industry or for the average consumer. Additionally, using protein sources not used regularly in commercial egg replacers, e.g., microalgae, faba bean, and lentil, could provide progress for closing the gap in the egg replacer market. 

Summarising all determined attributes, none of the egg replacers achieved comparable results to the control cakes, displaying a significant gap in the current development state of commercial egg replacers.

## Figures and Tables

**Table 1:** Ingredients of egg replacers used to replace whole egg powder in pound cake.

Egg Replacer	Ingredients
Egg replacer 1	potato starch, tapioca starch, calcium carbonate, citric acid, vegetable gum methylcellulose
Egg replacer 2	potato flour, tapioca flour, cream of tartar, xanthan gum, methylcellulose
Egg replacer 3	potato starch, tapioca flour, baking soda, psyllium husk fibre
Egg replacer 4	corn starch, mono- and diglycerides of fatty acids, xanthan gum, rice flour, pea protein
Egg replacer 5	tapioca starch, potato starch, pea protein, baking powder with pure tartar, guar gum
Egg replacer 6	corn starch, turmeric, gelling agent, chickpea flour, baking soda, tartaric acid
Egg replacer 7	whole grain buckwheat flour, tapioca starch, corn starch, psyllium seed, Himalayan black salt, vanilla flavour
Egg replacer 8	sweet lupine flour, locust bean gum, cornflour, flax protein, sunflower protein
Egg replacer 9	corn starch, potato protein, pea protein, lupin flour, maltodextrin, rock salt, turmeric powder, black salt, xanthan gum, locust bean gum flour, ground white pepper
Egg replacer 10	chia seeds, chickpea

**Table 2:** Nutritional compositions of egg replacers and control ingredient used in this study (per 100 g).

Per 100 g	C1	C2	C3	R1	R2	R3	R4	R5	R6	R7	R8	R9	R10
Energy [kcal]	595	155	147	306	30	300	480	339	358	338	324	356	357
Carbohydrates [g]	3.5	0	0	72.4	8.3	80	58	76.1	40.3	74	14	67.5	57.2
- quantities of which comprised sugars [g]	0	0	0	0.9	0.1	0	0	1.5	9.4	0.3	6.6	1.2	0
Total fibre [g]	0	0	0	2.8	2.6	10	6.1	-	11.4	-	37	0.7	28.6
Protein [g]	48	12.5	12.6	0	0.1	0	2	7.3	18.7	6.7	30	19.5	28.6
Fat [g]	38	8.9	10.8	0.1	0.1	0	25	0.7	5.1	1.3	8.3	0.3	7.2
- saturated fat [g]	-	3.6	3.2	0	0.05	0	25	0.1	0.65	0.2	1.2	0	0
- trans fat [g]	-	-	-	0	-	0	0	-	-	0	-	-	0
Sodium [g]	-	0.14	0.25	0.01	0.01	3.2	0.5	0.7	<0.1	0.7	0.04	2.1	0

C1: whole egg powder, C2: fresh eggs, C3: liquid pasteurised eggs. Egg replacers are numbered from R1 to R10 (Table 1).

**Table 3:** Pound cake formulations with egg and egg replacers; all values are given based on flour (%).

	C1	C2	C3	R1	R2	R3	R4	R5	R6	R7	R8	R9	R10
Biscuit flour	100	100	100	100	100	100	100	100	100	100	100	100	100
Sucrose	100	100	100	100	100	100	100	100	100	100	100	100	100
Salt	0.4	0.4	0.4	0.4	0.4	0.4	0.4	0.4	0.4	0.4	0.4	0.4	0.4
Baking powder	3.5	3.5	3.5	3.5	3.5	3.5	3.5	3.5	3.5	3.5	3.5	3.5	3.5
Fat	80.3	80.3	80.3	80.3	80.3	80.3	80.3	80.3	80.3	80.3	80.3	80.3	80.3
Whole egg powder	27.2	-	-	-	-	-	-	-	-	-	-	-	-
Fresh egg	-	109.4	-	-	-	-	-	-	-	-	-	-	-
Pasteurised liquid egg	-	-	109.4	-	-	-	-	-	-	-	-	-	-
Replacer	-	-	-	18.1	9.8	27.2	36.2	15.7	24.4	31.1	9.8	17.3	20.5
Water	81.9	-	-	90.9	99.2	81.9	72.8	93.7	85.0	78.0	99.2	91.7	88.6

C1: whole egg powder, C2: fresh eggs, C3: liquid pasteurised eggs. Egg replacers are numbered from R1 to R10 (Table 1).

**Table 4:** Effect of added egg replacer on properties of cake batter (structure changes during heating, viscosity of the cake batter). Values are presented as averages ± standard deviations.

	C1	C2	C3	R1	R2	R3	R4	R5	R6	R7	R8	R9	R10
Ti [°C]	82.47 ± 0.39 ^e,f,g^	82.18 ± 0.83 ^f,g,h^	80.67 ± 0.63 ^h^	81.12 ± 0.71 ^f,g^	84.58 ± 0.50 ^c,d^	85.62 ± 0.45 ^a,b,c^	86.50 ± 0.49 ^a,b^	84.92 ± 1.96 ^b,c,d^	86.30 ± 0.89 ^a,b^	86.72 ± 0.27 ^a^	82.87 ± 0.39 ^e,f^	86.02 ± 0.64 ^a,b,c^	83.97 ± 0.74 ^d,e^
Apparent viscosity [Pa∙s]	18.08 ± 1.01 ^d,e^	10.40 ± 1.38 ^f^	10.71 ± 0.66 ^f^	13.92 ± 1.85 ^e,f^	11.87 ± 1.14 ^f^	29.40 ± 1.87 ^b^	83.38 ± 11.79 ^a^	11.33 ± 1.12 ^f^	22.38 ± 1.34 ^c,d^	27.86 ± 1.17 ^b,c^	18.09 ± 1.21 ^d,e^	12.43 ± 0.84 ^f^	32.68 ± 2.57 ^b^

C1: whole egg powder, C2: fresh eggs, C3: liquid pasteurised eggs. Egg replacers are numbered from R1 to R10 (Table 1). No significant difference occurred between values in the same column with the same lowercase letter (*p* < 0.05).

**Table 5:** Physical properties and textures of the different pound cakes. Values are presented as the averages ± standard deviations.

Cake	Physical Properties		Texture				
	Bake Loss [%]	Water Activity [aw]	Springiness	Resilience	Cohesiveness	Chewiness [J]	Adhesiveness [J]
C1	9.39 ± 0.62 ^f,g^	0.765 ± 0.064 ^c^	0.91 ± 0.01 ^a,b^	0.24 ± 0.01 ^a,b^	0.62 ± 0.01 ^b^	4.55 ± 0.30 ^b^	−0.010 ± 0.004 ^a^
C2	9.38 ± 0.41 ^f,g^	0.864 ± 0.018 ^a,b^	0.93 ± 0.01 ^a^	0.29 ± 0.01 ^a^	0.68 ± 0.01 ^a^	4.81 ± 0.41 ^a,b^	−0.006 ± 0.004 ^a^
C3	10.79 ± 0.31 ^b,c,d^	0.884 ± 0.016 ^a^	0.92 ± 0.02 ^a,b^	0.28 ± 0.04 ^a^	0.65 ± 0.05 ^a^	3.86 ± 0.47 ^c,d,e^	−0.007 ± 0.004 ^a^
R1	11.26 ± 0.49 ^a,b^	0.879 ± 0.031 ^a^	0.86 ± 0.02 ^c,d,e^	0.19 ± 0.02 ^b,c,d^	0.50 ± 0.04 ^c,d^	3.09 ± 0.44 ^f,g^	−0.040 ± 0.029 ^a,b^
R2	10.02 ± 0.33 ^d,e,f^	0.809 ± 0.040 ^b,c^	0.85 ± 0.02 ^c,d,e^	0.20 ± 0.02 ^b,c,d^	0.51 ± 0.03 ^c^	5.28 ± 0.89 ^a^	−0.152 ± 0.092 ^c^
R3	11.68 ± 0.41 ^a^	0.872 ± 0.016 ^a^	0.84 ± 0.02 ^d,e,f^	0.20 ± 0.02 ^b,c^	0.51 ± 0.02 ^c^	2.21 ± 0.32 ^h^	−0.038 ± 0.018 ^a,b^
R4	6.93 ± 0.41 ^h^	0.857 ± 0.017 ^a,b^	0.88 ± 0.02 ^b,c^	0.13 ± 0.02 ^d^	0.40 ± 0.04 ^g,h^	3.28 ± 0.98 ^e,f,g^	−0.124 ± 0.095 ^c^
R5	10.88 ± 0.19 ^b,c^	0.897 ± 0.017 ^a^	0.84 ± 0.04 ^c,d,e,f^	0.18 ± 0.04 ^c,d^	0.47 ± 0.05 ^d,e^	4.33 ± 0.44 ^b,c^	−0.117 ± 0.054 ^c^
R6	9.79 ± 0.09 ^e,f^	0.872 ± 0.019 ^a^	0.80 ± 0.12 ^f^	0.20 ± 0.17 ^b,c^	0.39 ± 0.04 ^h^	4.29 ± 0.89 ^b,c,d^	−0.117 ± 0.048 ^c^
R7	10.59 ± 0.23 ^b,c,d^	0.881 ± 0.012 ^a^	0.86 ± 0.02 ^c,d,e^	0.15 ± 0.01 ^c,d^	0.43 ± 0.02 ^f,g^	2.77 ± 0.44 ^g,h^	−0.040 ± 0.023 ^a,b^
R8	10.21 ± 0.68 ^c,d,e^	0.884 ± 0.018 ^a^	0.82 ± 0.09 ^e,f^	0.21 ± 0.15 ^b,c^	0.43 ± 0.03 ^f,g^	3.72 ± 0.59 ^d,e^	−0.101 ± 0.043 ^b,c^
R9	11.16 ± 0.27 ^a,b^	0.877 ± 0.018 ^a^	0.85 ± 0.03 ^c,d,e^	0.18 ± 0.01 ^c,d^	0.46 ± 0.03 ^e,f^	3.54 ± 0.70 ^e,f^	−0.045 ± 0.027 ^a,b^
R10	8.78 ± 0.27 ^g^	0.869 ± 0.030 ^a^	0.88 ± 0.02 ^b,c,d^	0.13 ± 0.01 ^d^	0.43 ± 0.03 ^f,g^	4.21 ± 0.82 ^b,c,d^	−0.279 ± 0.187 ^d^

C1: whole egg powder, C2: fresh eggs, C3: liquid pasteurised eggs. Egg replacers are numbered from R1 to R10 (Table 1). No significant difference occurred between values in the same column with the same lowercase letter (*p* < 0.05).

**Table 6:** Appearance and colour of the different pound cakes. Values are presented as the averages ± standard deviations.

Cake	Physical Properties				Colour	
	Number of Cells	Number of Holes	Cell Diameter [mm]	Cell Elongation	ΔE (Crust)	ΔE (Crumb)
C1	3099 ± 259 ^a^	2.72 ± 1.35 ^b^	1.91 ± 0.20 ^b^	1.48 ± 0.01 ^c,d^	0.00 ^c^	0.00 ^e^
C2	2798 ± 263 ^b^	0.46 ± 0.80 ^d^	1.89 ± 0.14 ^b^	1.48 ± 0.01 ^c,d^	4.10 ± 0.96 ^a,b,c^	3.04 ± 0.61 ^d,e^
C3	2520 ± 190 ^c^	0.88 ± 0.66 ^c,d^	2.12 ± 0.16 ^b^	1.65 ± 0.28 ^a^	2.14 ± 2.40 ^b,c^	2.28 ± 1.81 ^e^
R1	1490 ± 176 ^g,h^	0.43 ± 0.47 ^d^	4.97 ± 6.30 ^a^	1.44 ± 0.02 ^d^	2.62 ± 2.72 ^b,c^	9.19 ± 4.96 ^c,d^
R2	1880 ± 120 ^d,e^	1.33 ± 0.79 ^c^	1.89 ± 0.36 ^b^	1.53 ± 0.02 ^b,c^	2.70 ± 1.53 ^b,c^	13.67 ± 2.05 ^b,c^
R3	1897 ± 83 ^d^	0.35 ± 0.41 ^d^	2.44 ± 0.29 ^b^	1.47 ± 0.02 ^c,d^	10.85 ± 1.41 ^a^	16.43 ± 0.79 ^b,c^
R4	3064 ± 238 ^a^	5.23 ± 2.03 ^a^	1.49 ± 0.14 ^b^	1.58 ± 0.03 ^a,b^	2.85 ± 0.99 ^b,c^	4.57 ± 1.33 ^d,e^
R5	1455 ± 131 ^h^	0.26 ± 0.51 ^d^	2.99 ± 0.80 ^b^	1.46 ± 0.02 ^c,d^	4.18 ± 3.16 ^a,b,c^	18.36 ± 0.87 ^b^
R6	1490 ± 96 ^g,h^	0.53 ± 0.51 ^c,d^	2.89 ± 0.90 ^b^	1.48 ± 0.03 ^c,d^	1.57 ± 1.95 ^b,c^	12.80 ± 0.73 ^b,c^
R7	1639 ± 130 ^f,g^	0.41 ± 0.50 ^d^	2.69 ± 0.36 ^b^	1.44 ± 0.02 ^d^	2.26 ± 1.63 ^b,c^	18.01 ± 0.20 ^b^
R8	1403 ± 159 ^h^	0.57 ± 0.58 ^c,d^	2.94 ± 0.62 ^b^	1.45 ± 0.02 ^d^	4.94 ± 3.29 ^a,b,c^	15.55 ± 0.59 ^b^
R9	1693 ± 147 ^f^	0.63 ± 0.59 ^c,d^	2.20 ± 0.27 ^b^	1.45 ± 0.02 ^d^	5.89 ± 4.18 ^a,b,c^	3.09 ± 2.98 ^d,e^
R10	1720 ± 162 ^e,f^	1.02 ± 0.87 ^c^	2.43 ± 0.34 ^b^	1.50 ± 0.04 ^c,d^	7.95 ± 1.96 ^a,b^	29.10 ± 2.13 ^a^

C1: whole egg powder, C2: fresh eggs, C3: liquid pasteurised eggs. Egg replacers are numbered from R1 to R10 (Table 1). No significant difference occurred between values in the same column with the same lowercase letter (*p* < 0.05).

## Data Availability

Data is contained within the article.
